# Cohesive-strength homogenisation model of porous and non-porous materials using linear comparison composites and application

**DOI:** 10.1038/s41598-020-60152-w

**Published:** 2020-02-25

**Authors:** Hyuk Lee, Vanissorn Vimonsatit, Wai Yeong Huen, Priyan Mendis, Kasun Shanaka Kristombu Baduge

**Affiliations:** 1 School of Civil Engineering and Mechanical Engineering, Curtin University, Western Australia, Australia; 2 School of Engineering, Civil Engineering, Macquarie University, New South Wales, Australia; 3 Department of Infrastructure Engineering, University of Melbourne, Victoria, Australia

**Keywords:** Civil engineering, Computational methods

## Abstract

An estimation of the strength of composite materials with different strength behaviours of the matrix and inclusion is of great interest in science and engineering disciplines. Linear comparison composite (LCC) is an approach introduced for estimating the macroscopic strength of matrix-inclusion composites. The LCC approach has however not been expanded to model non-porous composites. Therefore, this paper is to fill this gap by developing a cohesive-strength method for modelling frictional composite materials, which can be porous and non-porous, using the LCC approach. The developed cohesive-strength homogenisation model represents the matrix and inclusion as a two-phase composite containing solids and pores. The model is then implemented in a multiscaling model in which porous cohesive-frictional solids intermix with each other at different scale levels classified as micro, meso and macro. The developed model satisfies an upscaling scheme and is suitable for investigating the effects of the microstructure, the composition, and the interface condition of the materials at micro scales on the macroscopic strength of the composites. To further demonstrate the application of the developed cohesive-strength homogenisation model, the cohesive-strength properties of very high strength concrete are determined using instrumented indentation, nonlinear limit analysis and second-order cone programming to obtain material properties at different scale levels.

## Introduction

Development of composite materials is on-going to meet the demand for high standard of performance and in-service reliability. It has been pointed out that one of the most important factors that controls the elastic and plastic fields of composite materials is their local properties^1–5^. An estimation of the effective mechanical properties of composite materials based on microstructural properties and a suitable homogenisation model is of great interest in science and engineering disciplines^6,7^. Several homogenisation models have been developed based on continuum micromechanics which have enabled predicting macroscopic strength criteria for composite materials from considering the strength behaviour of the materials^3,4,6–9^. In the field of strength homogenisation, Dormieux *et al*.^8^ have extended the model with cohesive strength attributes. They have derived the strength domain of the materials in a form of cohesive-frictional solids with porosity. Furthermore, Ortega *et al*.^9^ introduced the strength domain for cohesive-frictional composite materials with porosity based on an application of a linear comparison composite (LCC) approach^6,7^. LCC is a homogenisation model of heterogeneous composites. The basic principle of LCC approach is to evaluate the plastic dissipation potential of nonlinear composites for selected linear comparison composites with a similar underlying microstructure^9^. Furthermore, a general type of second-order LCC has been introduced by Castaneda^6,7^. The LCC approach has however not been expanded for modelling non-porous composite materials. Therefore, this work is to develop a cohesive-strength method for modelling frictional composite materials, which are porous and non-porous, based on the LCC approach. The developed cohesive-strength homogenisation model is then implemented in a multiscaling scheme which is extended from existing formulation to investigate the effects of the material’s microstructure, composition, and interface condition, on the macroscopic strength. A multiscaling approach satisfies an upscaling scheme (micro to macro) in which porous cohesive-frictional solids intermix with each other.

The rest of the paper is organised as follows: Section 2 describes the cohesive-strength homogenisation model based on a second-order homogenisation and LCC approach. Section 3 proposes the concept of a multiscale-link approach for determining properties of materials at micro, meso and macro scales. Section 4 demonstrates the application of the proposed approach on very high strength concrete to investigate the cohesive-strength properties at different scales.

## Cohesive-Strength Homogenisation Model

A second-order homogenisation approach and strength homogenisation method have been introduced by Castaneda^6,7^ and Ortega *et al*.^9^, respectively. In order to develop a cohesive-strength homogenisation model of porous or non-porous composite, we recall briefly concepts of strength homogenisation method. Consider a representative volume element (RVE) of a composite material, Ω = Ω_1_ ∪ Ω_2_ where the Ω_1_ and Ω_2_ are the domains occupied by the two-phases, the subscripts 1 and 2 denote each phase domain. The material behaviour of a generic material phase *i* in the composite material is assumed to be characterised by local convex failure criterion ${f}_{i}\left({\boldsymbol{\sigma }}\right)\le 0\ \;\iff \;\ {\boldsymbol{\sigma }}\in {G}_{i}$, where *G*_*i*_ is a local convex strength domain. At the plastic collapse, each material phase no longer stores external work thus the external work is dissipated through plastic flow. This leads to the maximum dissipation capacity at the plastic collapse of each material phase which can be defined by the support function ${\pi }_{i}\left({\boldsymbol{d}}\right)={\sup }_{{\boldsymbol{\sigma }}\in {G}_{i}}\left[{\boldsymbol{\sigma }},{\boldsymbol{d}}\right]$, where ***d*** is the microscopic strain rate corresponding to the velocity field ***v***^9,10^. According to Hill lemma^11^, the upper bound theorem and plastic flow rule with relevant kinematic admissible (K.A) field, the microscopic dissipation function *π*_*i*_ is linked within the counterpart at the scale of RVE. It leads to the macroscopic dissipation function Π_*hom*_ with respect to the macroscopic stress **Σ** and the strain rate ***D*** as:

1 $${\Pi }_{hom}\left({\boldsymbol{D}}\right)={\sup }_{{\boldsymbol{\Sigma }}\in {G}_{hom}}\left[{\boldsymbol{\Sigma }},{\boldsymbol{D}}\right]$$

where *G*_*hom*_ is the boundary of the macroscopic domain^10^. According to the dual definition, the stress located at the intersection of *G*_*hom*_ can be expressed by:

2 $${\boldsymbol{\Sigma }}\in {G}_{hom}\ \;\iff \;\ {\boldsymbol{\Sigma }}:{\boldsymbol{D}}\le {\Pi }_{hom}\left({\boldsymbol{D}}\right)\quad {\rm{where}}\forall {\boldsymbol{D}};\quad {\boldsymbol{\Sigma }}=\frac{\partial {\Pi }_{hom}\left({\boldsymbol{D}}\right)}{\partial \left({\boldsymbol{D}}\right)}$$

The position vector of the macroscopic scale in Ω is denoted by x that leads to the local state equation as:

3 $${\boldsymbol{\sigma }}\left(x\right)={\mathbb{C}}\left(x\right):{\boldsymbol{d}}\left(x\right)+{\boldsymbol{\tau }}\left(x\right)\quad {\rm{where}}\forall x\in \Omega $$

For an isotropic material, the elastic stiffness ${\mathbb{C}}$ and prestress tensor *τ* of each phase can be expressed as:

4 $$\begin{array}{ccc}{{\mathbb{C}}}_{i}\left(x\right)= & 3{k}_{i}{\mathbb{J}}+2{g}_{i}{\mathbb{K}}\quad \left(x\in {\Omega }_{i}\right)\quad {\rm{where}}\,i=1,2 & \\ {\boldsymbol{\tau }}\left(x\right)= & {\tau }_{i}{\bf{1}}\quad \left(x\in {\Omega }_{i}\right)\quad {\rm{where}}\,i=1,2 & \end{array}$$

where *k*_*i*_ and *g*_*i*_ are the bulk and shear modulus of the corresponding phase. With classic linear micromechanics, the macroscopic stress equation can be represented as:

5 $${\boldsymbol{\Sigma }}={{\mathbb{C}}}_{hom}:{\boldsymbol{D}}+{{\boldsymbol{\tau }}}_{hom}$$

where ${{\mathbb{C}}}_{hom}$ and ***τ***_*hom*_ are macroscopic elastic stiffness tensor and prestress, respectively. Considering two-phase composite without discontinuity (variables associated with subscript 1 and 2), the strain rate energy function of an isotropic material can be expressed by:

6 $$\begin{array}{ccc}\psi \left({D}_{v},{D}_{d}\right)= & \frac{1}{2}{k}_{hom}{D}_{v}^{2}+2{g}_{hom}{D}_{d}^{2}+\beta \left({\tau }_{1}-{\tau }_{2}\right){D}_{v}+\frac{\gamma {\left({\tau }_{1}+{\tau }_{2}\right)}^{2}}{2{g}_{1}} & \\  & \beta =\frac{{k}_{hom}-{k}_{2}}{{k}_{1}-{k}_{2}};\qquad \gamma =\frac{{g}_{1}}{{k}_{1}-{k}_{2}}\left(\beta -{f}_{1}\right) & \end{array}$$

where ${D}_{v}={\rm{tr}}\left({\boldsymbol{D}}\right)$ and ${D}_{d}=\sqrt{\left(1/2\right)\Delta :\Delta }$ with $\Delta ={\boldsymbol{D}}-{\rm{tr}}\left({\boldsymbol{D}}\right){\bf{1}}$. According to the linear homogenisation scheme^8^, macroscopic bulk modulus *k*_*hom*_ and shear modulus *g*_*hom*_ can be represented by dimensional function ${\mathscr{K}}{g}_{1}={k}_{hom}$ and ${\mathscr{M}}{g}_{1}={g}_{hom}$, respectively. The Mori-Tanaka estimation of the effective behaviour of a two-phase composite material can be illustrated implicitly with a matrix-inclusion scheme^12^. In the present work, the microstructure of the two-phase composite will be represented by a spherical inclusion. The results from the use of linear micromechanics^13^ to obtain the homogenised bulk and shear modulus can be found in Part A of the Supplementary Information. The classic yield function of Drucker-Prager is defined by the mean stress ${\sigma }_{m}=1/3{\rm{tr}}\left({\boldsymbol{\sigma }}\right)$ and stress invariance ${\sigma }_{d}=\sqrt{\left(1/2\right){\boldsymbol{s}}:{\boldsymbol{s}}}$ with ***s*** = ***σ*** − *σ*_*m*_**1** as:

7 $$f\left({\boldsymbol{\sigma }}\right)={\sigma }_{m}+\alpha {\sigma }_{d}-c\le 0$$

where *α* and *c* are the Drucker-Prager friction coefficient and cohesion describing the intrinsic strength of each phase, respectively, i.e., $\alpha  < \sqrt{3}$/2 which is corresponding to Morh-Coulomb friction angle of 90°. With the application of stationary conditions through the material dissipation function of each phase, the degree of nonlinearity for the Drucker-Prager condition of each phase can be^9^:

8 $$\begin{array}{lll}{Y}_{i}= & \frac{{B}_{i}}{2{g}_{i}}\left[\frac{{\left({S}_{i}-{\tau }_{i}\right)}^{2}}{2{A}_{i}}-1\right]\quad {\rm{where}}\,i=1,2; & \\  & {B}_{i}={A}_{i}{\alpha }_{i};\quad {A}_{i}\to 0;\quad {S}_{i}={c}_{i}/{\alpha }_{i};\qquad \frac{{k}_{i}}{{g}_{i}}=\frac{{A}_{i}}{{B}_{i}}=\frac{1}{{\alpha }_{i}^{2}} & \end{array}$$

Therefore, the macroscopic strain rate energy density Π_*hom*_ for the upper bound solution can be found by employing the generated expression of the strain rate energy function in Eq.  (6) and nonlinearity function Eq.  (8) with the stationary conditions as:

9 $${\Pi }_{hom}\left({\boldsymbol{D}}\right)={{\rm{stat}}}_{{{\mathbb{C}}}_{i},{{\boldsymbol{\tau }}}_{i}}\ \left[\psi \left({\boldsymbol{D}}\right)+{\sum }_{i}{f}_{i}{Y}_{i}\right]\quad {\rm{with}}\quad \frac{\partial {\Pi }_{hom}\left({\boldsymbol{D}}\right)}{\partial {{\mathbb{C}}}_{i}}=0;\quad \frac{\partial {\Pi }_{hom}\left({\boldsymbol{D}}\right)}{\partial {{\boldsymbol{\tau }}}_{i}}=0;$$

where *f*_*i*_ is the volume fraction of each phase. The macroscopic strain rate energy density function can be obtained by rearranging Eqs. 1 to (8), which yields:

10 $${\Pi }_{hom}\left({\boldsymbol{D}}\right)={S}_{hom}{D}_{v}-sng\left(\rho \right)\sqrt{{A}_{hom}{D}_{v}^{2}+4{B}_{hom}{D}_{d}^{2}}\quad {\rm{subject}}\ {\rm{to}}\quad {A}_{hom}{D}_{v}^{2}+4{B}_{hom}{D}_{d}^{2}\ge 0$$

**Figure 1:**
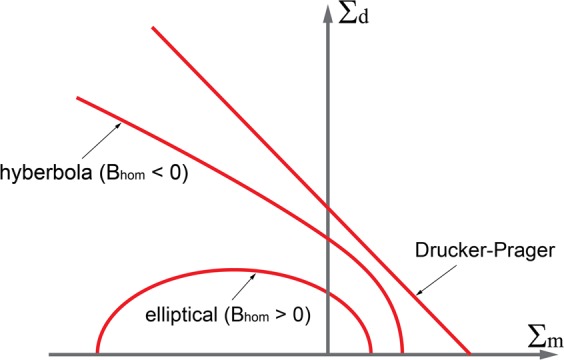
Cohesive-strength yield strength criterion.

The constraint in Eq. 10 ensures the validity of the macroscopic strain rate energy density function, and the shape of the yield function will depend on the *ρ* sign, hyperbolic when *ρ* > 0, and elliptical when *ρ* < 0, as shown in Fig. 1. In order to derive the dual definition of the strength domain represented by Eq. 2, the macroscopic yield strength criterion can be obtained as:

11 $$F\left({\boldsymbol{\Sigma }}\right)=sng\left({B}_{hom}\right)\left[\frac{{\left({\Sigma }_{m}-{S}_{hom}\right)}^{2}}{{A}_{hom}}+\frac{{\Sigma }_{d}^{2}}{{B}_{hom}}\right]\le 0$$

where ${\Sigma }_{m}=1/3{\rm{tr}}\left({\boldsymbol{\Sigma }}\right)$ and ${\Sigma }_{d}=\sqrt{\left(1/2\right){\boldsymbol{S}}:{\boldsymbol{S}}}$ with ***S*** = Σ − Σ_*m*_**1**. The shape of the macroscopic yield strength criterion depends on the sign of *B*_*hom*_, that is, the elliptical strength criterion for the positive value of *B*_*hom*_, and the hyperbolic strength criterion for the negative value of *B*_*hom*_, as shown in Fig. 1. This proposed yield strength criterion can be used to estimate the strength of composite materials at macroscopic scale.

## Multiscaling Cohesive-Strength

**Figure 2:**
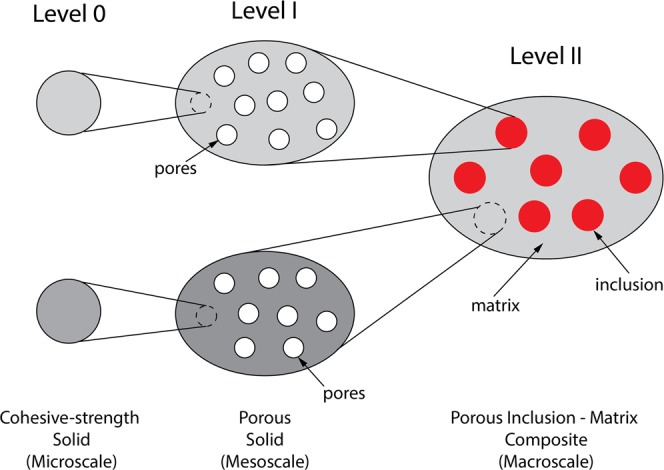
Systematic multiscale-link structure of composite material.

A general class of heterogeneous materials such as cementitious or geological materials is composed of particles which forms a porous composite material at the micrometre scale^8^. Let’s consider a composite material at three scale levels: a cohesive-strength solid (Level 0), porous solid (Level I), and porous inclusion-matrix (Level II), as shown in Fig. 2. Implementation of the proposed cohesive-strength yield criterion with LCC approach requires multiscale-link modelling in the strength homogenisation. The yield strength domain of the porous solid (Level I) is considered a cohesive-strength porous material in which the existence of elementary solid build block (Level 0) is present. In Level II, an RVE of Ω of the two-phase composite material has the inclusion phase surrounding the matrix phase as sub-index I and II, respectively. The systematic multiscale-link structure of the composite material as presented in Fig. 2 serves as a reference for implementing the cohesive-strength model in Eq. 11.

### Level II: porous inclusion-matrix composite

Consider a two-phase composite material with perfect adherence between interface at Level II as shown in Fig. 2, the first phase and second phase are cohesive frictional porous inclusion and matrix, respectively. In turn, the volume fraction of the matrix phase is characterised by *f*_1_ while the volume fraction of the inclusion phase *f*_2_ = 1 − *f*_1_. The scaling relation of the macroscopic strain rate energy density function between Level I and Level II can be expressed as:

12 $${{\mathbb{C}}}^{\left(I\right)}=\left\{\begin{array}{ll}{{\mathbb{C}}}_{1}= & 3{k}_{1}^{\left(I\right)}{\mathbb{J}}+2{g}_{1}^{\left(I\right)}{\mathbb{K}}\\ {{\mathbb{C}}}_{2}= & 3{k}_{2}^{\left(I\right)}{\mathbb{J}}+2{g}_{2}^{\left(I\right)}{\mathbb{K}}\end{array}\right.;\quad {{\boldsymbol{\tau }}}^{\left(I\right)}=\left\{\begin{array}{ll}{\tau }_{1}= & {\tau }_{1}^{\left(I\right)}{\boldsymbol{1}}\\ {\tau }_{2}= & {\tau }_{2}^{\left(I\right)}{\boldsymbol{1}}\end{array}\right.;\quad {\rm{with}}\quad {k}^{\left(II\right)}={{\mathscr{K}}}^{\left(II\right)}{g}_{1}^{\left(I\right)};\quad {g}^{\left(II\right)}={{\mathscr{M}}}^{\left(II\right)}{g}_{1}^{\left(I\right)}$$

13 $${S}_{hom}^{\left(II\right)}=\frac{\zeta }{\xi };\quad {A}_{hom}^{\left(II\right)}=\frac{\kappa \omega }{{\xi }^{2}};\quad {B}_{hom}^{\left(II\right)}=\frac{-{{\mathscr{M}}}^{\left(II\right)}\omega }{\xi };\quad {\rho }^{\left(II\right)}=\frac{\omega }{\xi }$$

with

$$\begin{array}{rcl}\xi  & = & {r}_{g}^{\left(I\right)}{{\alpha }_{1}^{\left(I\right)}}^{2}{{\alpha }_{2}^{\left(I\right)}}^{2}{f}_{1}-{r}_{g}^{\left(I\right)}{{\alpha }_{1}^{\left(I\right)}}^{2}{{\alpha }_{2}^{\left(I\right)}}^{2}{f}_{1}^{2}+2{\gamma }^{\left(II\right)}{{\alpha }_{1}^{\left(I\right)}}^{2}{f}_{1}-2{\gamma }^{\left(II\right)}{r}_{g}^{\left(I\right)}{{\alpha }_{2}^{\left(I\right)}}^{2}{f}_{1}+2{\gamma }^{\left(II\right)}{r}_{g}^{\left(I\right)}{{\alpha }_{2}^{\left(I\right)}}^{2}\\ \omega  & = & {f}_{1}{\gamma }^{\left(II\right)}{r}_{g}^{\left(I\right)}{({\alpha }_{1}^{\left(I\right)}{c}_{2}^{\left(I\right)}-{\alpha }_{2}^{\left(I\right)}{c}_{1}^{\left(I\right)})}^{2}\left({f}_{1}-1\right)\\ \zeta  & = & {\alpha }_{1}^{\left(I\right)}{\alpha }_{2}^{\left(I\right)}{\beta }^{\left(II\right)}{f}_{1}{r}_{g}^{\left(I\right)}({\alpha }_{1}^{\left(I\right)}{c}_{2}^{\left(I\right)}-{\alpha }_{2}^{\left(I\right)}{c}_{1}^{\left(I\right)})({f}_{1}-1)\\ \kappa  & = & {{\mathscr{K}}}^{\left(II\right)}{r}_{g}^{\left(I\right)}{{\alpha }_{1}^{\left(I\right)}}^{2}{{\alpha }_{2}^{\left(I\right)}}^{2}{f}_{1}^{2}-{{\mathscr{K}}}^{\left(II\right)}{r}_{g}^{\left(I\right)}{{\alpha }_{1}^{\left(I\right)}}^{2}{{\alpha }_{2}^{\left(I\right)}}^{2}{f}_{1}+2{{\alpha }_{1}^{\left(I\right)}}^{2}{{\beta }^{\left(II\right)}}^{2}{f}_{1}-2{{\mathscr{K}}}^{\left(II\right)}{\gamma }^{\left(II\right)}{{\alpha }_{1}^{\left(I\right)}}^{2}{f}_{1}\\  &  & -2{r}_{g}{{\alpha }_{1}^{\left(I\right)}}^{2}{{\beta }^{\left(II\right)}}^{2}{f}_{1}+2{r}_{g}{{\alpha }_{2}^{\left(I\right)}}^{2}{{\beta }^{\left(II\right)}}^{2}++2{{\mathscr{K}}}^{\left(II\right)}{\gamma }^{\left(II\right)}{r}_{g}^{\left(I\right)}{{\alpha }_{2}^{\left(I\right)}}^{2}{f}_{1}-2{{\mathscr{K}}}^{\left(II\right)}{\gamma }^{\left(II\right)}{r}_{g}^{\left(I\right)}{{\alpha }_{2}^{\left(I\right)}}^{2}\\ {r}_{g}^{\left(I\right)} & = & \frac{{g}_{1}^{\left(I\right)}}{{g}_{2}^{\left(I\right)}};\quad {\beta }^{\left(II\right)}=\frac{{k}^{\left(II\right)}-{k}_{2}^{\left(I\right)}}{{k}_{1}^{\left(I\right)}-{k}_{2}^{\left(I\right)}};\quad {\gamma }^{\left(II\right)}=\frac{{g}_{1}^{\left(I\right)}}{{k}_{1}^{\left(I\right)}-{k}_{2}^{\left(I\right)}}({\beta }^{\left(II\right)}-{f}_{1})\end{array}$$

where ${\mathbb{J}}=\left(1/3\right){\delta }_{ij}{\delta }_{kl}$ and ${\mathbb{K}}={\mathbb{I}}-{\mathbb{J}}$ with fourth-order identity tensor ${\mathbb{I}}$. With the dual definition in Eq. 2, the cohesive-strength yield criterion in Level II can be expressed by:

14 $${F}_{hom}^{\left(II\right)}\left({\boldsymbol{\Sigma }}\right)=sng({B}_{hom}^{\left(II\right)})\left[\frac{{({\Sigma }_{m}-{S}_{hom}^{\left(II\right)})}^{2}}{{A}_{hom}^{\left(II\right)}}+\frac{{\Sigma }_{d}^{2}}{{B}_{hom}^{\left(II\right)}}\right]\le 0$$

Similarly, the macroscopic frictional coefficient ${\alpha }^{\left(II\right)}$ and cohesion ${c}^{\left(II\right)}$ in Level II can be expressed by:

15 $${\alpha }^{\left(II\right)}=\sqrt{-sgn\left({\rho }^{\left(II\right)}\right)\frac{{B}_{hom}^{\left(II\right)}}{{A}_{hom}^{\left(II\right)}}};\quad {c}^{\left(II\right)}=\sqrt{\frac{{B}_{hom}^{\left(II\right)}\left({A}_{hom}^{\left(II\right)}-{{S}_{hom}^{\left(II\right)}}^{2}\right)}{{A}_{hom}^{\left(II\right)}}}$$

### Level I: porous solid

In this section, the mesoscopic cohesive-strength yield criterion of a two-phase composite material formed by pores (voids) and solid frictional matrix phase is established. The volumetric description at this level involves the solid packing density *η*, i.e., pore void *ϕ* = 1 − *η*. According to Eq.  (4), the distribution of the elastic stiffness and prestress of porous solid can be described as:

16 $${{\mathbb{C}}}^{\left(0\right)}=\left\{\begin{array}{ll}{{\mathbb{C}}}_{1}= & 3{k}_{1}^{\left(0\right)}{\mathbb{J}}+2{g}_{1}^{\left(0\right)}{\mathbb{K}}\\ {{\mathbb{C}}}_{2}= & 0\end{array}\right.;\quad {{\boldsymbol{\tau }}}^{\left(0\right)}=\left\{\begin{array}{ll}{\tau }_{1}= & {\tau }_{1}^{\left(0\right)}{\bf{1}}\\ {\tau }_{2}= & 0\end{array}\right.;\quad {\rm{with}}\quad {k}^{\left(I\right)}={{\mathscr{K}}}^{\left(I\right)}{g}_{1}^{\left(0\right)};\quad {g}^{\left(I\right)}={{\mathscr{M}}}^{\left(I\right)}{g}_{1}^{\left(0\right)}$$

Therefore, by substituting the limits, *α*_2_ → 0, *c*_2_ → 0, *r*_*g*_ → 0, *f*_2_ → 0, and *f*_1_ → *η* in Eq. 13, the cohesive-strength yield criterion of porous solid becomes:

17 $$\begin{array}{ccc}{S}_{hom}^{\left(I\right)} & = & \frac{{{\mathscr{K}}}^{\left(I\right)}{\alpha }^{\left(0\right)}{c}^{\left(0\right)}\eta }{2{{\mathscr{K}}}^{\left(I\right)}{{\alpha }^{\left(0\right)}}^{2}-\eta };\quad {A}_{hom}^{\left(I\right)}=\frac{{{\mathscr{K}}}^{\left(I\right)}{{c}^{\left(0\right)}}^{2}{\eta }^{2}\left(\eta -{{\mathscr{K}}}^{\left(I\right)}{{\alpha }^{\left(0\right)}}^{2}\right)}{{\left(\eta -2{{\mathscr{K}}}^{\left(I\right)}{{\alpha }^{\left(0\right)}}^{2}\right)}^{2}}\\ {B}_{hom}^{\left(I\right)} & = & \frac{{{\mathscr{M}}}^{\left(I\right)}{c}^{\left(0\right)}\eta \left(\eta -{{\mathscr{K}}}^{\left(I\right)}{{\alpha }^{\left(0\right)}}^{2}\right)}{\eta -2{{\mathscr{K}}}^{\left(I\right)}{{\alpha }^{\left(0\right)}}^{2}};\quad {\rho }^{\left(I\right)}=\frac{{{c}^{\left(0\right)}}^{2}\eta \left(\eta -{{\mathscr{K}}}^{\left(I\right)}{{\alpha }^{\left(0\right)}}^{2}\right)}{2{{\mathscr{K}}}^{\left(I\right)}{{\alpha }^{\left(0\right)}}^{2}-\eta }\end{array}$$

In other words, Eq. 13 is reduced to Eq.  (17) to present the solution for the porous solid support function ${\Pi }_{hom}^{\left(I\right)}$, which is found to be the same as that of Ortega *et al*.^9^. It should be noted that $2{{\mathscr{K}}}^{\left(I\right)}{{\alpha }^{\left(0\right)}}^{2}-\eta  > 0$ provides a hyperbolic cohesive- strength criterion, while $2{{\mathscr{K}}}^{\left(I\right)}{{\alpha }^{\left(0\right)}}^{2}-\eta  < 0$ corresponds to an elliptical cohesive-strength yield criterion. Based on the dual definition of the strength domain presented in Eq. 2, the macroscopic yield strength criterion can then be derived as:

18 $${F}_{hom}^{\left(I\right)}\left({\boldsymbol{\Sigma }}\right)=sng({B}_{hom}^{\left(I\right)})\left[\frac{{({\Sigma }_{m}-{S}_{hom}^{\left(I\right)})}^{2}}{{A}_{hom}^{\left(I\right)}}+\frac{{\Sigma }_{d}^{2}}{{B}_{hom}^{\left(I\right)}}\right]\le 0$$

To retrieve the Drucker-Prager yield criterion, the macroscopic frictional coefficient ${\alpha }^{\left(II\right)}$ and cohesion ${c}^{\left(II\right)}$ in in Level I can then be obtained as:

19 $${\alpha }^{\left(I\right)}=\sqrt{-sgn\left({\rho }^{\left(I\right)}\right)\frac{{B}_{hom}^{\left(I\right)}}{{A}_{hom}^{\left(I\right)}}};\quad {c}^{\left(I\right)}=\sqrt{\frac{{B}_{hom}^{\left(I\right)}\left({A}_{hom}^{\left(I\right)}-{{S}_{hom}^{\left(I\right)}}^{2}\right)}{{A}_{hom}^{\left(I\right)}}}$$

## Application of Cohesive-Strength Homogenisation

The application of the proposed cohesive-strength homogenisation model is demonstrated in this section with a focus on the investigation of the properties of very high strength concrete (VHSC) (>100 MPa). The two main phases of VHSC are aggregate and matrix phases. High strength concrete (HSC) (50–100 MPa) and VHSC have been used increasingly in the construction industry due to its inherent performance characteristics. High compressive strength, high elastic modulus, very low permeability, low deformations are some of the contributing factors to the increasing uses^14^. SC and VHSC are very brittle in nature compared with the normal strength concrete (NSC), and the damage and fracture behaviour of HSC and VHSC are considerably different from the fracture behaviour of NSC^14–16^. Two mixtures, M1 and M2, are used to achieve VHSC with a water to binder ratio of 0.22. It is assumed that nano-silica is able to fill nano-sized gaps between the materials and therefore enhances the strength and performance of the mixtures. The high-range water reducing, slump retention, and viscosity modifying admixture have been used to retain the slump, workability, and avoid bleeding of the self-compacting and segregation. Two different types of coarse aggregate, basalt (M1) and granite (M2), are used in order to determine cohesive-strength properties with the mixture contents as shown in Table 1. The uniaxial compressive test and measurement of elastic modulus has been conducted on cylindrical concrete samples with the diameter of 100 mm and the height of 200 mm. The uniaxial compressive strength of M1 and M2 are obtained as 160 MPa and 134 MPa at 90 days curing ages, respectively. The values of the elastic modulus of M1 and M2 mixture are 52 GPa and 47 GPa, respectively.

**Table 1:** Mix design of VHSC.

Materials	M1 (kg)	M2 (kg)	Unit weight (kg/m^3^)
Cement	500		3110
Fly ash	52		2290
Slag	187		2860
Silica fume	60		2180
Coarse Aggregate	1280	1180	Basalt: 2940
			Granite: 2710
Fine aggregate	300	300	Sand: 2610

**Figure 3:**
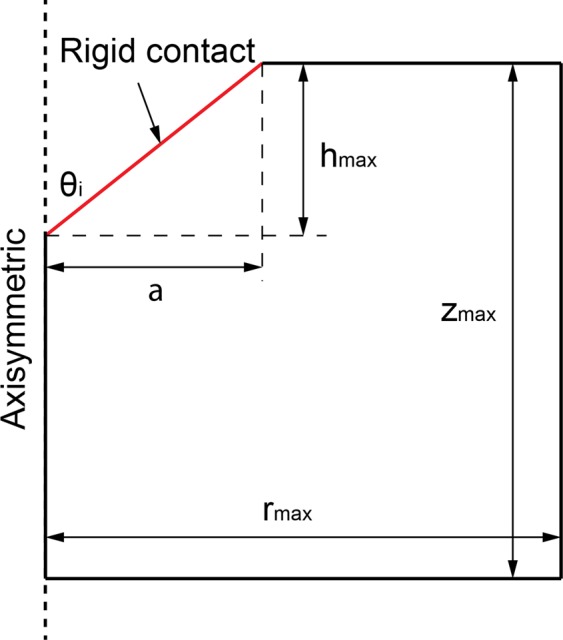
Model of indentation test for SOCP limit analysis.

In this section, the methodology for evaluating the materials properties based on the proposed cohesive-strength homogenisation model is demonstrated. Firstly, the cohesive-strength homogenisation model in Level I is applied for the continuum discretisation of instrumented indentation solution. The instrumented indentation is a well-known technique that has emerged for determining mechanical properties^17,18^. Recently, the instrumented indentation makes it possible to test the structure of porous materials with the characteristic size of porosity much smaller than the maximum indentation depth^19^. Bobko *et al*.^20^ developed an indentation hardness response with the developed strength homogenisation model by Ortega *et al*.^9^. They used a limit analysis solver to predict the indentation hardness which was nominalised by solid cohesion as a function of varying solid friction coefficient. In this paper, a similar approach is adopted to investigate the applicability of the developed cohesive-strength homogenisation model, based on the second-order cone programming (SOCP)^11^. Consider the indentation test of a rigid contact into a cylindrical composite material with polar coordinates *r*, *θ*, and *z* as shown in Fig. 3.

The work rate provides a rigid contact to composite material (assumed frictionless contact) during testing process as:

20 $$dW=P\dot{h}={\int }_{\Omega }{\boldsymbol{\Sigma }}:{\boldsymbol{D}}d\Omega $$

**Figure 4:**
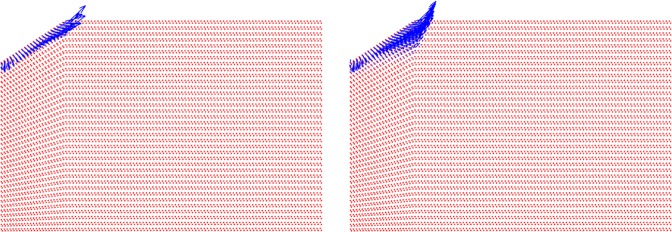
The effect of packing density on velocity field ${\alpha }^{\left(0\right)}=0.3$: *η* = 0 (left) and *η* = 0.5 (right).

**Figure 5:**
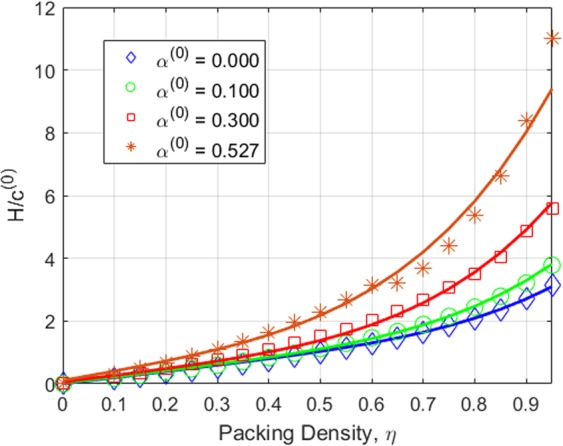
Dimensional function ${H}^{\left(I\right)}$/${c}^{\left(0\right)}$ from the discretisation of indentation solutions.

**Figure 6:**
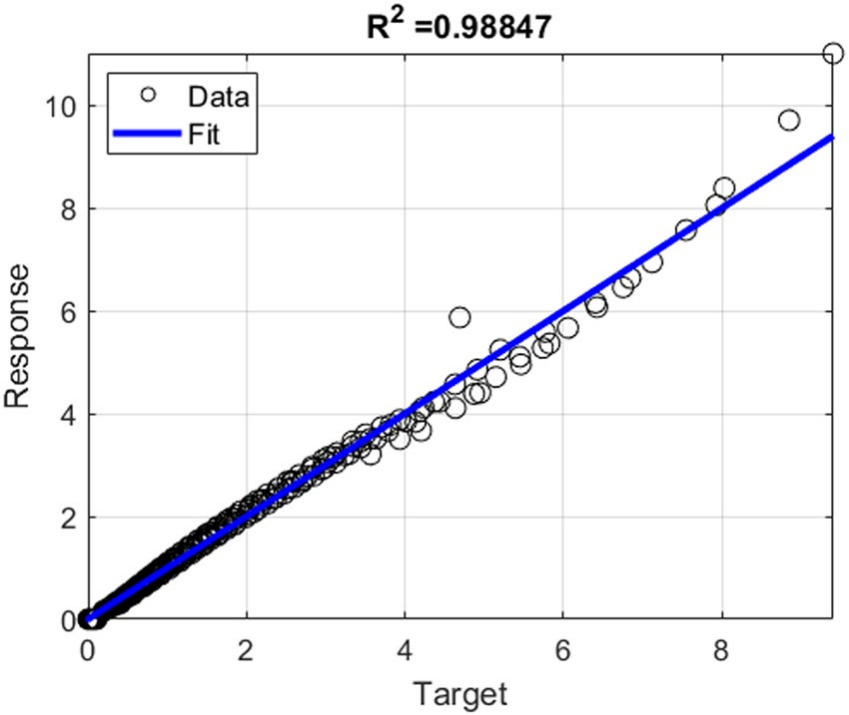
Correction coefficient of Level I cohesive-strength model.

With the combination of Eq. 10, the dimensional function in the form of the indentation hardness H to cohesion c ratio, with the upper bound solution of the cohesive-strength homogenisation can be expressed by:

21 $$\begin{array}{ccc} &  & \frac{{H}^{\left(I\right)}}{{c}^{\left(0\right)}}=\frac{1}{{A}_{c}\dot{h}}{\min }_{{\boldsymbol{U}}{\rm{k}}.{\rm{a}}{\boldsymbol{D}}}{\int }_{\Omega }\left(\frac{{S}_{hom}^{\left(I\right)}}{{c}^{\left(0\right)}}\right){D}_{v}-sng\left(\frac{{\rho }^{\left(I\right)}}{{c}^{\left(0\right)}}\right)\sqrt{\left(\frac{{A}_{hom}^{\left(I\right)}}{{{c}^{\left(0\right)}}^{2}}\right){D}_{v}^{2}+4\left(\frac{{B}_{hom}^{\left(I\right)}}{{{c}^{\left(0\right)}}^{2}}\right){D}_{d}^{2}}d\Omega \\  &  & \ \ {\rm{s}}.\,{\rm{t}}\left\{\begin{array}{l}\forall \left(r,\theta ,z\right)\in {A}_{c}\\ \left(r,\theta ,z\right)\to \infty ;U=0\\ \left(\frac{{A}_{hom}^{\left(I\right)}}{{{c}^{\left(0\right)}}^{2}}\right){D}_{v}^{2}+4\left(\frac{{B}_{hom}^{\left(I\right)}}{{{c}^{\left(0\right)}}^{2}}\right){D}_{d}^{2}\ge 0\end{array}\right.\end{array}$$

where *U* is the velocity field, $${A}_{c}=\pi {h}_{max}^{2}$$$${\tan }^{2}{\theta }_{i}$$ is the contact area of the indentation test when using an equivalent conical Berkovich tip with *θ*_*i*_ = 70.3 degree. The displacement rate $$\dot{h}$$ of the rigid indenter tip in the *z* direction is fixed as one. The dimensional function in Eq.  (21) consists of the key unknowns, *S*_*hom*_, *A*_*hom*_, and *B*_*hom*_ developing the relation between the cohesive-strength responses of the composite material. In determining the dimensional function, a design of experimental (DOE) approach^18^ is adopted considering variables such as *α* and *η* (more details are provided in Part B of the Supplementary Information). An advanced implementation of a continuum discretisation into finite elements together with nonlinear composites and SOCP techniques have been recognised for the upper bound theorem^6,7^. In particular, the approach used in this study is the numerical implementation of the discretised dissipation of the cohesive-strength criterion ($$\alpha  < \sqrt{3}$$/2) in the form of a second-order cone optimisation problem which can be solved by MOSEK optimisation algorithm^21^ available in MATLAB^22^. In the implementation of the finite element discretisation, the triangle shape function in the axisymmetric condition shown in Fig. 3 has been modelled. The radius of the sample to contact ratio, *r*_*max*_/*a*, and the thickness to the indentation ratio, *z*_*max*_/*h*, are set to 5; and the radius of the sample to element size ratio, *r*_*max*_/*L*_*i*_ = 100; the equivalent conical Berkovich tip apex angle, *θ*_*i*_, of 70.3 degree is set with the frictionless contact between the rigid contact and the sample. Eq.  (21) can be solved as a SOCP optimisation problem, in which the dimensional functions, as presented in Eq.  (17), are calculated for all the combination of the parameters outlined in the DOE, Table B, given the Supplementary Information. Figures 4 and 5 show a typical series of the velocity field and the relationship between various $${\alpha }^{\left(0\right)}$$ and the dimensional function $${H}^{\left(I\right)}/{c}^{\left(0\right)}$$, respectively. The solution of the SOCP problem, Eq.  (21), yields the discrete solution of the hardness to cohesion ratio which can be represented by a polynomial curve-fitting function for practical use of the results for Level I. The correction coefficient *R*^2^ and root mean square error (RMSE) have been used to evaluate the prediction of the regression quality. A comprehensive parametric study of 484 cases was then conducted to represent the range of the parameters of cohesive-strength responses. The results of the empirical correction of the curve fitting show a good agreement (*R*^2^ = 0.988 and RMSE = 0.1997) with the target values as shown Fig. 6. The obtained $${\alpha }^{\left(0\right)}$$, $${c}^{\left(0\right)}$$ and *eta* are of the specific interest when minimising the error between theoretical and experimental indentation hardness $${H}_{\exp }^{\left(I\right)}$$ results. The nonlinear optimisation algorithm is therefore performed to minimise the following quantity:

22 $${\min }_{{\alpha }^{\left(0\right)},{c}^{\left(0\right)},\eta }{\sum }_{i}^{n}{\left[{H}_{\exp }^{\left(I\right)}-{H}_{i}^{\left(I\right)}({\alpha }^{\left(0\right)},{c}^{\left(0\right)},{\eta }_{i})\right]}^{2}$$

**Figure 7:**
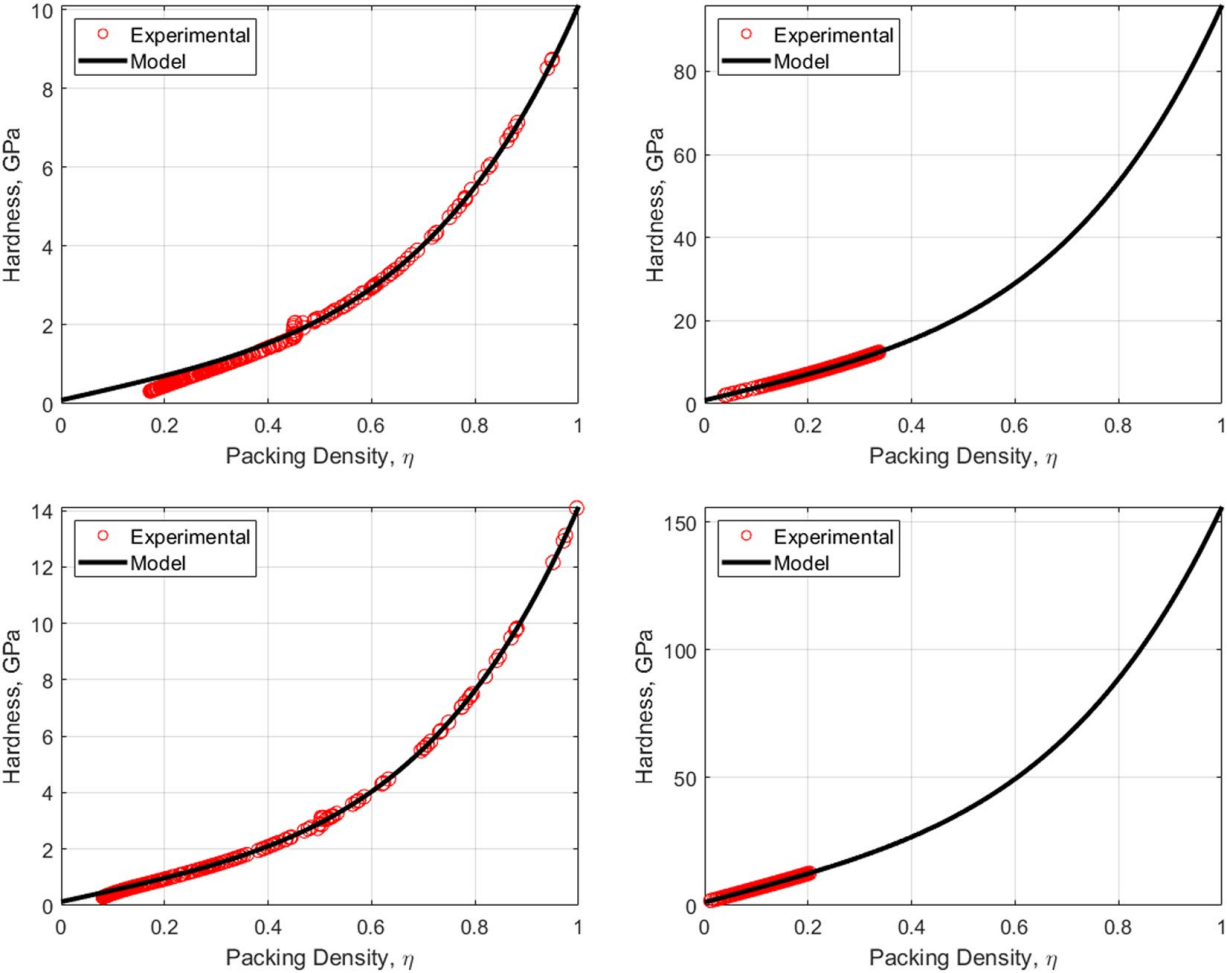
Scaling relation of indentation hardness to packing density of M1 matrix (Top left) and aggregate (Top right); M2 matrix (bottom left) and aggregate (bottom right).

It should be noted that the efficiency of the minimisation process depends on computational resources. The first application of the cohesive-strength homogenisation model in Level I focuses on the investigation of solid properties of aggregate and cementitious matrix of VHSC. Each specimen was cut using diamond saw to obtain 10mm cube core part after 90 days curing ages. It is important to reduce the surface roughness of specimens to obtain an accurate nanoindentation results because the specimen’s surface has a significant influence on the test results. In order to apply the continuum indentation model of an infinite half-space, the indentation depth must be larger than the surface roughness. Therefore, fine emery paper was used to grind all specimens to reduce the surface roughness then the specimens were polished using a suspension solution ranging 9.0 *μ*m to 0.1 *μ*m for 15 mins. This is achieved with the maximum indentation depth 500 nm of VHSC samples. Nanoindentation test was conducted on the three sets of cube specimens with XP BASIC testing mode with Keysight Nanoindentation G200; each set was specified around 300 indentation points on the cementitious matrix, fine sand and coarse aggregate using the Berkovich indenter. The hardness values of each indentation point then determined using our inverse algorithm^18^. Based on the indentation results, the hardness properties of solid at Level 0 and the properties of the material at Level I (with pores) are determined by Eq. 19. The results of the scaling relation of the indentation hardness to the packing density are illustrated in Fig. 7. The values determined at Level 0 and Level I of the packing density *η* of all samples are presented in Tables 2 and 3. The Level 0 (solid) properties of M1 and M2 matrices show that ${\alpha }^{\left(0\right)}$ = 0.660 and 0.626, ${c}^{\left(0\right)}$ = 0.600 and 1.042 GPa, ${k}^{\left(0\right)}$ = 8.913 and 8.333 GPa, ${g}^{\left(0\right)}$ = 3.887 and 3.220 GPa, and that of the Level I properties are: *η*_*avg*_ = 0.264 and 0.213, ${\alpha }_{avg}^{\left(I\right)}$ = 0.263 and 0.346, ${c}_{avg}^{\left(I\right)}$ = 0.123 and 0.170 GPa, ${k}_{avg}^{\left(I\right)}$ = 1.040 and 0.703 GPa, and ${g}_{avg}^{\left(I\right)}$ = 0.072 and 0.084 GPa, respectively. Similarly, the variation of the Level 0 responses of M1 and M2 aggregates captured by the cohesive-strength with the indentation test results are: ${\alpha }^{\left(0\right)}$ = 0.706 and 0.775, ${c}^{\left(0\right)}$= 5.836 and 8.086 GPa, ${k}^{\left(0\right)}$ = 2217.2 and 4465.6 GPa, ${g}^{\left(0\right)}$ = 1107.1 and 2688.3 GPa, and that of Level 1 responses are: *η*_*avg*_ = 0.244 and 0.129, ${\alpha }_{avg}^{\left(I\right)}$ = 0.199 and 0.166, ${c}_{avg}^{\left(I\right)}$ = 1.094 and 0.774 Pa, ${k}_{avg}^{\left(I\right)}$ = 253.764 and 277.629 GPa, and ${g}_{avg}^{\left(I\right)}$ = 10.077 and 7.695 GPa, respectively. he present results show that even though M1 and M2 matrices have the same mix design, Level 0 (solid) properties are not consistent to each other. It is believed that the development of calcium silicate hydrated (CSH) which is one of major hydration products of cementitious matrix contributing to the primary source of nanometre-scale elastic modulus degradation^13,18^. In fact that the nanostructure of CSH is still not known, forms poorly crystalline during the hydration of the cementitious matrix and characterised by extensive atomic imperfection and structural variations at nanometre scale^23^. Despite inconsistent Level 0 (solid) properties, the characteristic of Level I properties are consistent. The uniaxial compressive strength Σ_*UCS*_ represents the stress state in Level II macroscopic strength domain obtained by substituting Σ_*m*_ = − 1 ⁄ 3Σ_*UCS*_ and ${\Sigma }_{d}=\left(\sqrt{3}/3\right){\Sigma }_{UCS}$ in Eq. 14. Evaluating the cohesive-strength criterion for Σ_*UCS*_ requires the values of the inclusion-matrix factors ${\mathscr{K}}$ and ${\mathscr{M}}$. Two conditions are considered in this study, (i) perfect adherence in which inclusions and matrix are perfectly bonded and (ii) the case of slip or nonfictional interface which characterised by a purely normal stress vector acting on the interface^9^ (more details are provided in Supplementary Information). The results of the cohesive-strength behaviour of M1 and M2 mixtures at Level II are presented in Table 4. The Level II properties of perfect adherence case are obtained as: M1, ${\alpha }^{\left(II\right)}$ = 0.019, ${c}^{\left(II\right)}$ = 0.094 GPa, ${k}^{\left(II\right)}$ = 49.105 GPa, ${g}^{\left(II\right)}$ = 0.017 GPa, and Σ_*UCS*_ = 162.288 MPa; M2, ${\alpha }^{\left(II\right)}$ = 0.017, ${c}^{\left(II\right)}$ = 0.073 GPa, ${k}^{\left(II\right)}$ = 34.488 GPa, ${g}^{\left(II\right)}$ = 0.010 GPa, and Σ_*UCS*_ = 125.553 MPa. The properties of the slip-interface case, are: M1, ${\alpha }^{\left(II\right)}$ = 0.012, ${c}^{\left(II\right)}$ = 0.060 GPa, ${k}^{\left(II\right)}$ = 47.674 GPa, ${g}^{\left(II\right)}$ = 0.007 GPa, and Σ_*UCS*_ = 103.686 MPa; M2, ${\alpha }^{\left(II\right)}$ = 0.011, ${c}^{\left(II\right)}$ = 0.047 GPa, ${k}^{\left(II\right)}$ = 33.908 GPa, ${g}^{\left(II\right)}$ = 0.004 GPa, and Σ_*UCS*_ = 81.141 MPa. The value of Σ_*UCS*_ of perfect adherence is close to the experimental uniaxial compressive strength ${\Sigma }_{UCS}^{EXP}$, with around 6% difference. The continuum discretisation into finite elements of nonlinear composites using SOCP techniques have been conducted to verify the results of the cohesive-strength behaviour of M1 and M2 mixture at Level II using Eq. 10. The radius of the sample *r*_*max*_ and the height *z*_*max*_ are set to 50 and 200, respectively; and the element size *L*_*i*_ = 2; with the frictionless contact between the rigid contact and the sample. The uniaxial strength can be determined from Σ_*UCS*_ = *P*∕*A*_*c*_ with the contact area of *A*_*c*_ = *πr*^2^. Combining Eqs. 10 and 20, the uniaxial compressive strength with the upper bound solution (kinematic assemble (k.a) velocity filed) of cohesive-strength homogenisation can be expressed by:

23 $$\begin{array}{ccc} &  & {\Sigma }_{UCS}=\frac{1}{{A}_{c}\dot{h}}{\min }_{{\boldsymbol{U}}{\rm{k}}.{\rm{a}}{\boldsymbol{D}}}{\int }_{\Omega }{S}_{hom}^{\left(II\right)}{D}_{v}-sng\left({\rho }^{\left(II\right)}\right)\sqrt{{A}_{hom}^{\left(II\right)}{D}_{v}^{2}+4{B}_{hom}^{\left(I\right)}{D}_{d}^{2}}d\Omega \\  &  & {\rm{s}}.\,{\rm{t}}\left\{\begin{array}{l}\forall \left(r,\theta ,z\right)\in {A}_{c}\\ \left(r,\theta ,z\right)\to \infty ;U=0\\ {A}_{hom}^{\left(II\right)}{D}_{v}^{2}+4{B}_{hom}^{\left(II\right)}{D}_{d}^{2}\ge 0\end{array}\right.\end{array}$$

**Table 2:** Level 0 (Solid) properties of VHSC.

	M1		M2	
	Matrix	Aggregate	Matrix	Aggregate
Friction coefficient ${\alpha }^{\left(0\right)}$	0.660	0.706	0.626	0.775
Cohesion ${c}^{\left(0\right)}$ (MPa)	0.600	5.836	1.042	8.086
Bulk modulus ${k}^{\left(0\right)}$ (GPa)	8.913	2217.2	8.333	4465.6
Shear modulus ${g}^{\left(0\right)}$ (GPa)	3.887	1107.1	3.220	2688.3

**Table 3:** Level I properties of VHSC.

	M1		M2	
	Matrix	Aggregate	Matrix	Aggregate
Average friction coefficient ${\alpha }_{avg}^{\left(I\right)}$	0.263	0.199	0.346	0.166
Average packing density *η*_*avg*_	0.264	0.244	0.213	0.129
Average cohesion ${c}_{avg}^{\left(I\right)}$ (MPa)	0.123	1.094	0.170	0.774
Average bulk modulus ${k}_{avg}^{\left(I\right)}$ (GPa)	1.040	253.76	0.703	277.62
Average shear modulus ${g}_{avg}^{\left(I\right)}$ (GPa)	0.072	10.077	0.084	7.695

**Table 4:** Level II uniaxial compressive strength of VHSC.

		M1	M2
Perfect adherence	Friction coefficient ${\alpha }^{\left(II\right)}$	0.019	0.017
	Cohesion ${c}^{\left(II\right)}$	0.094	0.073
	Bulk modulus ${k}^{\left(II\right)}$ (GPa)	49.105	34.448
	Shear modulus ${g}^{\left(II\right)}$ (GPa)	0.017	0.010
	Uniaxial compressive strength Σ_*UCS*_ (MPa)	162.288	125.553
	${\Sigma }_{UCS}/{\Sigma }_{UCS}^{EXP}$	0.985	1.067
Slip interface	Friction coefficient ${\alpha }^{\left(II\right)}$	0.012	0.011
	Cohesion ${c}^{\left(II\right)}$	0.060	0.047
	Bulk modulus ${k}^{\left(II\right)}$ (GPa)	47.674	33.908
	Shear modulus ${g}^{\left(II\right)}$ (GPa)	0.007	0.004
	Uniaxial compressive strength Σ_*UCS*_ (MPa)	103.686	81.141
	${\Sigma }_{UCS}/{\Sigma }_{UCS}^{EXP}$	1.543	1.651

**Figure 8:**
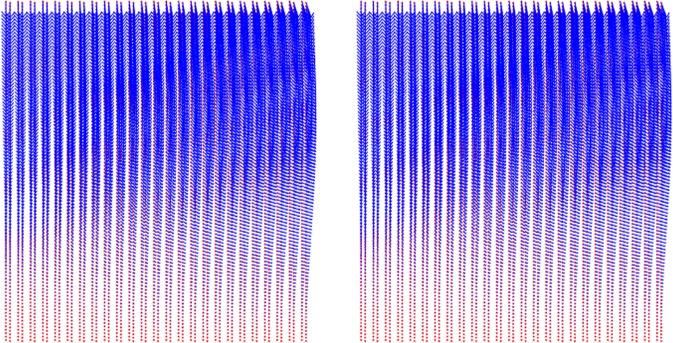
Dissipation capacity of velocity filed of M1 (left) and M2 (right) mixtures.

The results of the nonlinear limit analysis with SOCP problem of Σ_*UCS*_ presented in Eq. (23) are then obtained for a perfect adherence case, in this case, Σ_*UCS*_ = 170.2 and 131.7 MPa for M1 and M2 mixtures, respectively; and for a slip interface, Σ_*UCS*_ =108.9 and 85.2 MPa for M1 and M2 mixtures, respectively. Thus, a good agreement is observed between the theoretical and the experimental values. The results of the velocity field of the nonlinear limit analysis with SOCP problem of Level II are illustrated in Fig. 8.

## Conclusion

The proposed cohesive-strength homogenisation model enables predicting the behaviour of porous and non-porous two-phase composites. The fundamental idea behind the developed approach presented in this paper is that it is possible to assess the cohesive-strength behaviour of composite materials at different scale levels. The novelty of the present model is the incorporation of the two-phase nonlinear relationship of cohesive-strength composites based on a linear comparison composite (LCC) approach. The developed cohesive-strength model represents a multiscale-link relation of composite materials which incorporates the contribution of different phases for determining the effective mechanical properties. An application of the proposed model has been demonstrated on very high strength concrete (VHSC) materials which have two main phases, aggregate and matrix. A three-level multiscale conceptual model is formulated which makes it possible to determine microscale and macroscale properties of the materials. The proposed cohesive-strength model approach combines an instrumented indentation technique, a nonlinear limit analysis and a second-order cone programming method to quantitatively predict the strength of VHSC from micro to macro level. Overall, the proposed cohesive-strength model can be used to predict the mechanical properties of material phases at different scale levels.

## Supplementary information

Supplementary Information.
