# Supplementary material for: Cohesive-strength homogenisation model of porous and non-porous materials using linear comparison composites and application
Source: Sci Rep. 2020 Feb 25;10:3425. doi: 10.1038/s41598-020-60152-w (PMC7042320; doi:10.1038/s41598-020-60152-w)
Supplement: Supplementary file 1 — Supplementary Information. [file 41598_2020_60152_MOESM1_ESM.pdf]

Cohesive-strength homogenisation model of porous and non-porous materials using linear comparison composites and application

Hyuk Lee[a], Vanissorn Vimonsatit[a], Wai Yeong Huen, Priyan Mendis[b], and Kasun Shanaka Kristombu Baduge[b]

[a] School of Civil and Mechanical Engineering, Curtin University, Western Australia, Australia

[b] Department of Infrastructure Engineering, University of Melbourne, Victoria, Australia

A. Mori-Tanaka estimates

A.1 Perfectly Bonded

The effective elastic stiffness of a two-phase composite material in a perfect adhesion case can be estimated by [1]

$$\mathbb{C}_{hom} = [f_1\mathbb{C}_1 : \mathbb{A}_1 + f_2\mathbb{C}_2\mathbb{A}_2] : [f_1\mathbb{A}_1 + f_2\mathbb{A}_2]^{-1} \tag{A1}$$

where f_1 and f_2 are the volume fraction of matrix and inclusion phase, respectively. Considering that the main phase plays the role of a matrix with the elastic stiffness tensor $\mathbb{C}_1$, then the strain concentration tensor $\mathbb{A}$ of each phase can be expressed by:

$$\mathbb{A}_i = [\mathbb{I} + \mathbb{P}_1 : (\mathbb{C}_i - \mathbb{C}_1)]^{-1} \tag{A2}$$

with

$$\mathbb{P}_1 = \frac{1}{3k_1 + 4g_1}\mathbb{J} + \frac{3}{5g_1}\frac{k_1 + 2g_1}{3k_1 + 4g_1}\mathbb{K}$$

For a given composite (Level II), it is assumed that each phase has an isotropic elastic behaviour, then the effective bulk and shear are:

$$k_{hom} = \frac{4g_1k_2 + 3k_1k_2 + 4f_1k_1g_1 - 4f_1k_2g_1}{3k_1 - 3f_1k_1 + 3f_1k_2 + 4g_1}$$
$$g_{hom} = \frac{g_2(20g_1g_2 + 15g_1k_2 + 8f_2g_2^2 - 8f_2g_1g_2 + 9f_2g_2k_2 - 9f_2g_2k_2)}{15g_2k_2 - 12f_2g_2^2 + 20g_2^2 + 12f_2g_1g_2 - 6f_2g_2k_2 + 6f_2g_1k_2} \tag{A3}$$

Substitute the bulk modulus in terms of the shear modulus and the friction coefficient $k_i = g_i/\alpha_i^2$ into Eq (A3) with $r_g = g_1/g_2$, thus,

$$\mathcal{K} = \frac{k_{hom}}{g_1} = \frac{4\alpha_2^2 - 4\alpha_1^2f_1 + 4\alpha_2^2f_1r_g + 3}{3\alpha_1^2f_1 + 3\alpha_2^2r_g + 4\alpha_1^2\alpha_2^2r_g - 3\alpha_2^2f_1r_g}$$
$$\mathcal{M} = \frac{g_{hom}}{g_1} = \frac{9f_1r_g - 9f_1 - 8\alpha_1^2f_1 + 20\alpha_1^2 + 8\alpha_1^2f_1r_g + 15}{6f_1 + 15r_g - 6f_1r_g + 12\alpha_1^2f_1 + 20\alpha_1^2r_g - 12\alpha_1^2f_1r_g} \tag{A4}$$

For the porous solid case (Level I), the void inclusions $k_2 = 0$ and $g_2 = 0$, then

$$\mathcal{K} = \frac{k_{hom}}{g_1} = \frac{4f_1}{4\alpha_1^2 - 3f_1 + 3}$$

$$\mathcal{M} = \frac{g_{hom}}{g_1} = \frac{f_1(8\alpha_1^2 + 9)}{5(4\alpha_1^2 + 3) - 6(f_1 + 2\alpha_1^2 f_1)} \tag{A5}$$

A.2 Slippery Interfaces

In the elastic homogenisation relations, the inclusions are considered to be rigid at Level II. Thus, the derivation of the elastic homogenisation relations of the matrix with rigid inclusion of slipper interfaces link the bulk and shear modulus as [2, 3]:

$$\mathcal{K} = \frac{k_{hom}}{g_1} = \frac{4f_2 + 3\alpha_1}{3(1 - f_2)}$$

$$\mathcal{M} = \frac{g_{hom}}{g_1} = \frac{3(3f_2 + 2)\alpha_1 + 4f_1(2f_1 + 3)}{6(1 - f_2)(2 + \alpha_1)} \tag{A6}$$

B. Design of Experiment

Based on a design of experimental approach (DOE), the levels of parameters used for determining the dimensional functions are listed in Table B.

Table B Design of experiment for Level I

	$\alpha^{(0)}$	η
1	0	0
2	0.050	0.100
3	0.100	0.150
4	0.150	0.200
5	0.200	0.250
6	0.250	0.300
7	0.300	0.350
8	0.350	0.400
9	0.400	0.450
10	0.450	0.500
11	0.500	0.550
12	0.527	0.600

C. Hardness to Cohesion Response from Instrumented Indentation

$$\begin{aligned}\frac{H^{+}}{c^{(0)}} = {} & 0.0000009707{\alpha^{(0)}}^{5} + 13.0713{\alpha^{(0)}}^{4}\eta + 0.00000050979{\alpha^{(0)}}^{4} \\ & + 0.000060776{\alpha^{(0)}}^{3}\eta^{2} + 0.0000026542{\alpha^{(0)}}^{3}\eta \\ & + 0.00000030739{\alpha^{(0)}}^{3} + 8.0328{\alpha^{(0)}}^{2}\eta^{3} + 0.00000062112{\alpha^{(0)}}^{2}\eta^{2} \\ & + 0.0000010214{\alpha^{(0)}}^{2}\eta + 0.00000026416{\alpha^{(0)}}^{2} + 7.7369\alpha^{(0)}\eta^{4} \\ & + 0.00000021661\alpha^{(0)}\eta^{3} + 0.0000001493\alpha^{(0)}\eta^{2} \\ & + 0.000018566\alpha^{(0)}\eta + 0.20219\eta + 1.5888\eta^{5} + 0.0000001018\eta^{4} \\ & + 0.000000053079\eta^{3} + 0.000000056275\eta^{2} + 1.9648\eta \\ & + 0.000000048461\end{aligned} \quad \text{(C1)}$$

References

1. Zaoui, A., *Continuum Micromechanics: Survey.* Journal of Engineering Mechanics, 2002. **128**(8): p. 808-816.
2. Ortega, J.A., B. Gathier, and F.-J. Ulm, *Homogenization of cohesive-frictional strength properties of porous composites: Linear comparison composite approach.* Journal of Nanomechanics and Micromechanics, 2011. **1**(1): p. 11-23.
3. Lee, H., et al., *An Investigation of Nanomechanical Properties of Materials using Nanoindentation and Artificial Neural Network.* Scientific Reports, 2019. **9**(1): p. 13189.
